# Integrated Strategies of Diverse Feature Selection Methods Identify Aging-Based Reliable Gene Signatures for Ischemic Cardiomyopathy

**DOI:** 10.3389/fmolb.2022.805235

**Published:** 2022-03-01

**Authors:** Huafeng Song, Shaoze Chen, Tingting Zhang, Xiaofei Huang, Qiyu Zhang, Cuizhi Li, Chunlin Chen, Shaoxian Chen, Dehui Liu, Jiawen Wang, Yingfeng Tu, Yueheng Wu, Youbin Liu

**Affiliations:** ^1^ Department of Cardiology, Guangzhou Eighth People’s Hospital, Guangzhou Medical University, Guangzhou, China; ^2^ Department of Cardiology, Huanggang Central Hospital, Huanggang, China; ^3^ Department of Cardiology, The First Affiliated Hospital of Harbin Medical University, Harbin, China; ^4^ Guangdong Provincial Key Laboratory of South China Structural Heart Disease, Guangdong Cardiovascular Institute, School of Medicine, Guangdong Provincial People’s Hospital and Guangdong Academy of Medical Sciences, South China University of Technology, Guangzhou, China; ^5^ School of Forensic Medicine, Guizhou Medical University, Guiyang, China

**Keywords:** ischemic cardiomyopathy, gene signature, feature selection, TRMT5, mitochondrial membrane potential, apoptosis

## Abstract

**Objective:** Ischemic cardiomyopathy (ICM) is a major cardiovascular state associated with prominently increased morbidity and mortality. Our purpose was to detect reliable gene signatures for ICM through integrated feature selection strategies.

**Methods:** Transcriptome profiles of ICM were curated from the GEO project. Classification models, including least absolute shrinkage and selection operator (LASSO), support vector machine (SVM), and random forest, were adopted for identifying candidate ICM-specific genes for ICM. Immune cell infiltrates were estimated using the CIBERSORT method. Expressions of candidate genes were verified in ICM and healthy myocardial tissues *via* Western blotting. JC-1 staining, flow cytometry, and TUNEL staining were presented in hypoxia/reoxygenation (H/R)-stimulated H9C2 cells with TRMT5 deficiency.

**Results:** Following the integration of three feature selection methods, we identified seven candidate ICM-specific genes including ASPN, TRMT5, LUM, FCN3, CNN1, PCNT, and HOPX. ROC curves confirmed the excellent diagnostic efficacy of this combination of previous candidate genes in ICM. Most of them presented prominent interactions with immune cell infiltrates. Their deregulations were confirmed in ICM than healthy myocardial tissues. TRMT5 expressions were remarkedly upregulated in H/R-stimulated H9C2 cells. TRMT5 deficiency enhanced mitochondrial membrane potential and reduced apoptosis in H/R-exposed H9C2 cells.

**Conclusion:** Collectively, our findings identified reliable gene signatures through combination strategies of diverse feature selection methods, which facilitated the early detection of ICM and revealed the underlying mechanisms.

## Introduction

Ischemic cardiomyopathy (ICM) represents the main cause of deaths and acts as an independent factor of mortality among all cardiomyopathy diseases (Luczak et al., 2020). This disease has the features of diverse clinical manifestations and pathophysiological substrates (Gu et al., 2020). It is diagnosed in accordance with left ventricular dysfunctions following epicardial coronary artery diseases (Cabac-Pogorevici et al., 2020). Currently, therapeutic modalities focus on relief of symptoms, prevention of ICM progression, and improvement of survival and quality of life (Razeghian-Jahromi et al., 2021). Heart transplantation and ventricular assist device implantation are applied for treating patients with severe heart failure (Mori et al., 2019). Nevertheless, issues like donor shortage and dismal complications hinder the effects of previous therapeutic modalities. Thus, more effective therapeutic regimens are urgently developed.

Accumulated evidence demonstrates that numerous fetal and immediate-early genes present deregulation in ICM (Dang et al., 2020). However, genetic causes and molecular mechanisms underlying ICM progression remain partially clarified (Suffee et al., 2020). Evidence suggests that gene signatures exert critical roles in diverse diseases that depend on the leapfrog advance of sequencing technologies (Li et al., 2020). For instance, Dang et al. proposed deregulated genes in ICM and their upstream transcription factors (Dang et al., 2020). Wang et al. reported that transcriptome profile analyses uncovered PHLDA1 as a candidate molecular signature for ICM (Wang et al., 2018). Cao et al. identified key hub genes of ICM in accordance with bioinformatics analyses (Cao et al., 2021). Current studies concerning ICM are limited by inadequate biological specimens from ICM patients. Thus, specific gene expression that involves pathological conditions of ICM requires in-depth exploration based on multiple ICM specimens. In the present study, our combination strategies of diverse feature selection methods identified candidate gene signatures, which contributed to an in-depth understanding of the mechanisms and novel diagnostic and therapeutic targets of ICM.

## Materials and Methods

### Retrieval of Gene Expression Profiling

From the Gene Expression Omnibus (GEO) repository (https://www.ncbi.nlm.nih.gov/gds/), we harvested the transcriptome data relevant to ICM patients. Under screening, two reliable datasets were retrieved, including GSE5406 and GSE46224 datasets. In brief, microarray expression profiling of left ventricular myocardium from 108 advanced ICM patients and 16 non-failing controls was curated from the GSE5406 dataset as the training set (Hannenhalli et al., 2006). This dataset was on the basis of the platform of GPL96 [HG-U133A] Affymetrix Human Genome U133A Array. High-throughput sequencing data of 16 failing and 8 non-failing control hearts were retrieved from the GSE46224 dataset that was based on the GPL11154 Illumina HiSeq 2000 (*Homo sapiens*) platform (Yang et al., 2014). The GSE46224 dataset acted as the validation set.

### Analysis of ICM-Specific Genes

By using linear models for microarray data (limma) package, expression values were compared between ICM and control left ventricular myocardium tissues (Ritchie et al., 2015). The corresponding *p*-values of the gene symbols following t-tests were determined. Thereafter, adjusted *p* < 0.05 and |fold change (FC)|>1.5 were utilized as the filtrating criteria. Using the pheatmap package, ICM-specific genes were visualized into the heatmap.

### Functional Enrichment Analyses

Using the clusterProfiler package (Yu et al., 2012), Gene Ontology (GO) enrichment analyses were carried out. The biological process, cell component, and molecular function of ICM-specific genes were separately investigated. The same tool was also adopted for the enrichment analyses of Kyoto Encyclopedia of Genes and Genomes (KEGG) pathways.

### Analyses of Protein–Protein Interaction

Using the Search Tool for the Retrieval of Interacting Genes/Proteins (STRING; https://string-db.org/) (Szklarczyk et al., 2021), the interactions of proteins encoded by ICM-specific genes were analyzed. Thereafter, a PPI network was conducted using Cytoscape software (Doncheva et al., 2019). Moreover, Molecular Complex Detection (MCODE), a plug-in in Cytoscape (Bader and Hogue, 2003), was used to screen modules of the PPI network in accordance with degree cutoff = 2, node score cutoff = 0.2, k-core = 2, and max. depth = 100. Nodes and edges in the PPI networks separately meant the proteins encoded by ICM-specific genes and their interactions.

### Feature Selection of Candidate Genes

Feature selection algorithms including least absolute shrinkage and selection operator (LASSO) analyses, support vector machine (SVM), and random forest were separately conducted using the glmnet package (Engebretsen and Bohlin, 2019), e1071 package, and random forest package. Following the intersection of characteristic genes from three algorithms, candidate ICM-specific genes were identified. The diagnostic efficacy of this combination model was evaluated by receiver operating characteristic (ROC) curves, and the area under the curve (AUC) was measured for investigations of accuracy, sensitivity, and specificity.

### Estimation of Immune Cell Subpopulation Fractions

The abundance of 22 immune cell subpopulations in left ventricular myocardium samples from the GSE5406 dataset was quantified using Cell type Identification By Estimating Relative Subsets Of RNA Transcripts (CIBERSORT; http://cibersort.stanford.edu/) (Newman et al., 2015). This analytical tool provides an estimation of the proportions of immune cell subpopulations across mixed cell populations utilizing normalized expression profiles. The infiltrating immune cells were estimated using CIBERSORT included B cells, T cells, natural killer cells, macrophages, dendritic cells, eosinophils, and neutrophils. The “LM22.txt” matrix comprises the “signature matrix” of 547 genes. For running CIBERSORT, the packages of e1071, parallel and preprocessCore were adopted. The *p*-value was derived from this method for deconvoluting all specimens utilizing Monte Carlo sampling. With *p* < 0.05, specimens of the estimated abundance of immune cell subpopulations generated with CIBERSORT were of high accuracy. Finally, the proportions of immune cell subpopulations were inferred across diverse tissues.

### Cell Culture

The H9C2 cell line was purchased from Chinese Academy of Sciences. Cells were maintained in high-glucose DMEM containing 10% fetal bovine serum (FBS) and 100 U/mL of penicillin/streptomycin. They were grown in an incubator with 5% CO_2_ at 37°C.

### Real-Time Quantitative Polymerase Chain Reaction

Total RNA was extracted from H9C2 cell specimens with TRIzol, which was quantified and reversely transcribed into cDNA. RT-qPCR was carried out utilizing the QuantStudio 5 Real-Time PCR System (Applied Biosystems, United States) with the SuperScript IV One-Step RT-PCR System (Invitrogen, United States). The qPCR conditions were as follows: initial denaturation at 95°C lasting 3 min, 40 cycles of denaturation at 95°C lasting 15 s, annealing at 60°C lasting 30 s, and elongation at 72°C lasting 15 s. The final cycle included extension at 72°C for 3 min. RT-qPCR primers used are listed in Table 1. The 2^−ΔΔCt^ method was applied for quantification.

**TABLE 1:** Primer sequences used for RT-qPCR.

Gene	Sequence
ASPN	5′-CTC​TGC​CAA​ACC​CTT​CTT​TAG​C-3′
	5′-CGT​GAA​TAG​CAC​TGA​CAT​CCA​A-3′
TRMT5	5′-CGT​TGA​TTC​CAG​TAG​CTT​GGA​C-3′
	5′-CTG​TTT​CTG​GCA​TGG​TTG​AGA-3′
FCN3	5′-GCC​CTC​CCA​GTC​TTT​TGT​GAC-3′
	5′-CCT​GGA​GAG​TAA​GCT​GGT​GC-3′
LUM	5′- TAA​CTG​CCC​TGA​AAG​CTA​CCC-3′
	5′-GGA​GGC​ACC​ATT​GGT​ACA​CTT-3′
CNN1	5′-CTG​TCA​GCC​GAG​GTT​AAG​AAC-3′
	5′-GAG​GCC​GTC​CAT​GAA​GTT​GTT-3′
PCNT	5′-TCA​CTT​CCG​ACA​GAG​AAA​AAC​AA-3′
	5′-CCT​GGA​CAG​ACG​CAT​CGA​C-3′
HOPX	5′-GAG​ACC​CAG​GGT​AGT​GAT​TTG​A-3′
	5′-AAA​AGT​AAT​CGA​AAG​CCA​AGC​AC-3′
GAPDH	5′-GGA​GCG​AGA​TCC​CTC​CAA​AAT-3′
	5′-GGC​TGT​TGT​CAT​ACT​TCT​CAT​GG-3′

### Western Blotting

Tissues or H9C2 cells were homogenized through precooled RIPA lysis buffer plus 1% protease and phosphatase inhibitors. Thereafter, specimens were centrifuged at 12,000 g lasting 15 min at 4°C. The supernatant was harvested for adding loading buffer, and the extract was boiled at 100°C lasting 5 min. Through sodium dodecyl sulfate polyacrylamide gel electrophoresis (SDS-PAGE), the protein extract was separated. Protein specimens were transferred onto polyvinylidene fluoride (PVDF) membranes. Thereafter, the membranes were under incubation with 5% skim milk lasting 1 h. The primary antibodies targeting ASPN (1/3000; ab154404; Abcam, United States), TRMT5 (1/3000; 18255-1-AP; Proteintech, Wuhan, China), FCN3 (1/1000; 11867-1-AP; Proteintech, Wuhan, China), LUM (1/1000; 10677-1-AP; Proteintech, Wuhan, China), CNN1 (1/3000; 24855-1-AP; Proteintech, Wuhan, China), PCNT (1/2000; ab231958; Abcam, United States), HOPX (1/2000; 11419-1-AP; Proteintech, Wuhan, China), *β*-actin (1/3000; 20536-1-AP; Proteintech, Wuhan, China), Bcl-2 (1/2000; 12789-1-AP; Proteintech, Wuhan, China), Bax (1/5000; 50599-2-Ig; Proteintech, Wuhan, China), and GAPDH (1/20000; 60004-1-Ig; Proteintech, Wuhan, China) were added for incubation by the membranes at 4°C overnight. Then, the membranes were incubated with the secondary antibody (1/3000; 7074; Cell Signaling Technology, United States) for 1 h at room temperature. The bots were visualized utilizing the enhanced chemiluminescence (ECL) detection system as well as assessed using ImageJ software.

### Patients and Specimens

Three patients were diagnosed with ICM, and three healthy controls were recruited from Guangdong Provincial People’s Hospital in our study. Human left ventricular myocardial tissues were collected from the three ICM patients during cardiac transplantation. Meanwhile, three healthy left ventricular myocardial tissues as controls were obtained from unused donor hearts. The study complied with the Declaration of Helsinki. This study gained the approval of the Ethics Committee of Guangdong Provincial People’s Hospital (GDREC2016255H). The signed informed consent of each participant was required.

### Establishment of Cell Models

When H9C2 cells grew to 70–80% density, they were digested by trypsin with ethylenediaminetetraacetic acid (EDTA). Thereafter, the cells were seeded onto six-well plates. Hypoxia/reoxygenation (H/R) cellular model was established. In brief, H9C2 cells were maintained in glucose- and serum-free DMEM as well as exposed to hypoxia at 37°C in a hypoxic plastic chamber (5% CO_2_, 1% O_2_, and 94% N_2_) lasting 8 h. Thereafter, H9C2 cells were subjected to reoxygenation at 37°C in a normoxic incubator (95% air and 5% CO_2_) lasting 24 h.

### Transfections

For silencing TRMT5 gene expression, H9C2 cells were transfected by small interfering RNAs (siRNAs; GenePharma, Shanghai, China) in accordance with manufacturer’s instructions. In brief, in a mixture of 100 pmol siRNAs, 5.0 μL Lipofectamine™ 2000 (Thermo Fisher, United States), and 500 μL DMEM, they were incubated lasting 20 min at room temperature. Thereafter, the mixture was transferred onto the plate and incubated lasting 37°C. The culture medium was replaced by a fresh growth medium plus FBS. Cardiomyocytes were collected 24 h following transfections and subjected onto subsequent assays. The specificity or efficiency of TRMT5 deficiency was investigated with RT-qPCR and Western blotting. Scrambled siRNA acted as a negative control (si-NC). Herein, two specific TRMT5 siRNAs (si-TRMT5#1 and si-TRMT5#2) were utilized to silence TRMT5 expression.

### JC-1 Staining

JC-1 staining (Thermo Fisher, United States) was presented for investigation of mitochondrial membrane potential. H9C2 cells were incubated by a mixture of working solution of JC-1 dye and cell culture medium. The excess dye was removed by washing with JC-1staining buffer and rinsed with PBS three times. Thereafter, cardiomyocytes were investigated and acquired using a confocal laser scanning microscope. OD values of green fluorescence (490 nm/530 nm) and red fluorescence (525 nm/590 nm) were separately examined. Eventually, data were displayed as a red/green fluorescence ratio.

### Flow Cytometric Analyses

Flow cytometric analyses were carried out utilizing FITC-labeled recombinant Annexin V apoptosis detection kits (Beyotime, China). H9C2 cells were washed with PBS and reconstituted with coupling buffer. Thereafter, cardiomyocytes were incubated by 250 ng/ml Annexin V-FITC protecting from light at 4°C lasting 15 min. Cardiomyocytes were washed with PBS and resuspended with 190 µL coupling buffer. Then, cardiomyocytes were exposed to 10 µL propidium iodide (PI) lasting 5 min. Staining cardiomyocytes were assessed using an FACStar plus flow cytometer. Data analyses were carried out utilizing a BD BioSciences FACSCalibur flow cytometer.

### Terminal Deoxynucleotidyl Transferase dUTP Nick-End Labeling Assay

H9C2 cells were maintained onto the chamber sections (1 × 10^4^ cells/chamber). Apoptotic cardiomyocytes were examined with TUNEL staining utilizing *In Situ* Cell Death Detection Kits in accordance with the supplier’s specification. Images were captured using an Olympus BX41 fluorescence microscope. Image quantification was carried out as percentages of TUNEL-positive cardiomyocytes within the entire number of cardiomyocytes.

### Statistical Analyses

*p*-values under 0.05 indicated significant differences. Data were expressed as mean ± SD. R software (version 3.4.0; https://www.r-project.org/) and Bioconductor packages (http://www.bioconductor.org/) and GraphPad Prism software (version 8.0.1) were adopted for statistical analyses. Student’s t-test or Wilcoxon test was utilized for comparing the significant differences between two subgroups. One-way analyses of variance (ANOVA) and Tukey’s post hoc test were presented for comparing multiple subgroups. Pearson or Spearman correlation test was conducted for evaluation of the interactions between variables.

## Results

### Detection of ICM-specific Genes as well as Their Biological Significance

**FIGURE 1:**
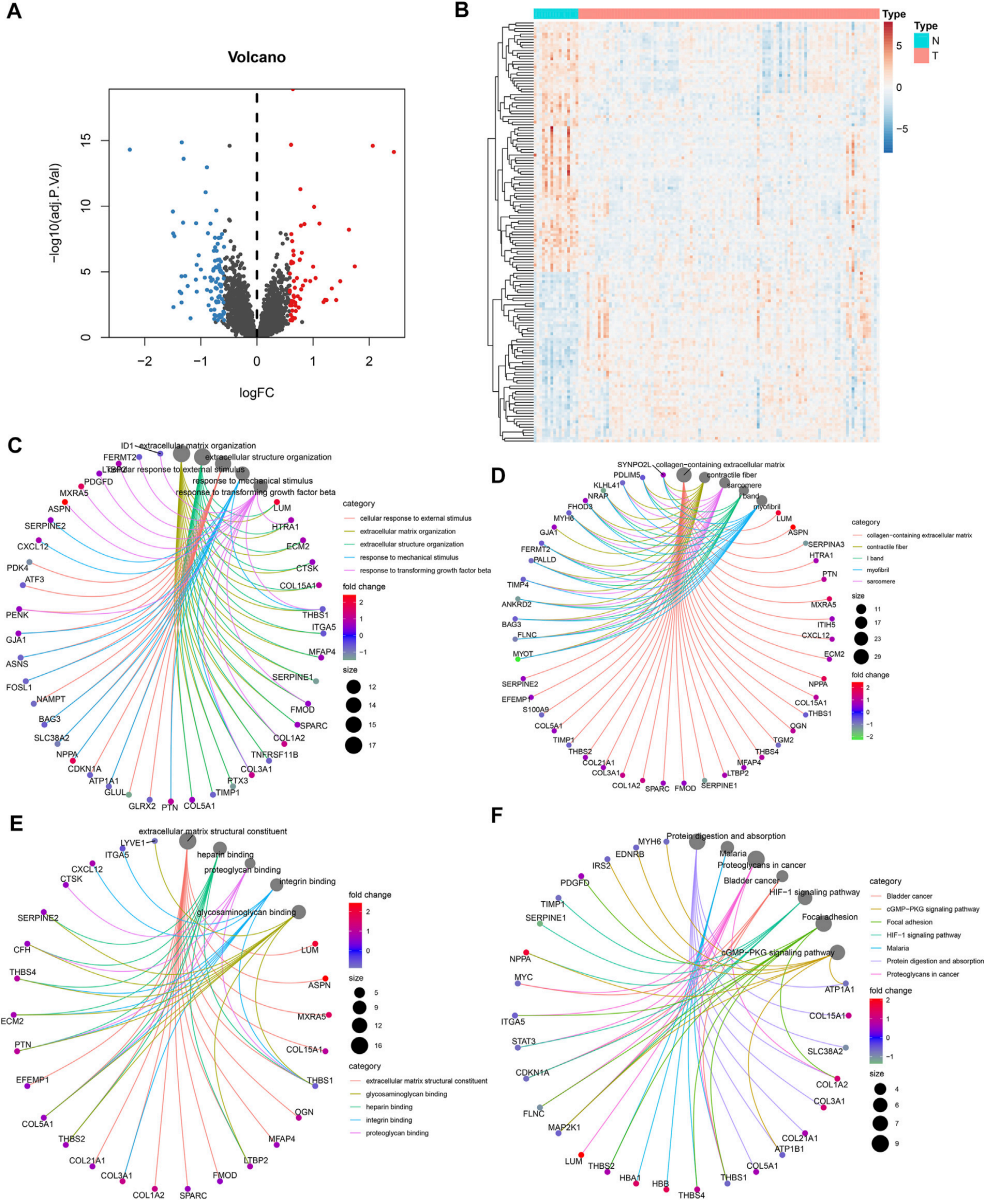
Visualization of ICM-specific genes and exploration of functional enrichment analyses. **(A)** ICM-specific genes screened through thresholds of FDR <0.05 and |fold change| >1.5 were visualized *via* volcano plots. Blue bots represent downregulated genes, while red bots represent upregulated genes in ICM. **(B)** Heatmap presents the expression patterns of ICM-specific genes in ICM and control left ventricular myocardium tissues. **(C–F)** Interaction networks show the most prominent **(C)** biological processes, **(D)** cellular components, **(E)** molecular functions, and **(F)** KEGG pathways enriched by ICM-specific genes.

Byemploying the transcriptome profiling of the left ventricular myocardium from 108 ICM patients and 16 non-failing controls in the GSE5406 dataset, we screened ICM-specific genes with the criteria of an FDR <0.05 and |fold change| >1.5. Consequently, 153 genes were specifically expressed in ICM (Figures 1A,B). Among them, 62 presented upregulation, while 91 presented downregulation in ICM left ventricular myocardium tissues (Tables 2, 3). Functional enrichment analyses conducted using the clusterProfiler package uncovered the ICM-specific genes-relevant biological significance and pathways. GO analyses demonstrated that the biological functions of ICM-specific genes were primarily associated with cellular response to external stimulus, extracellular matrix organization, extracellular structure organization, response to mechanical stimulus, and response to transforming growth factor beta (Figure 1C). In Figure 1D, cellular components of collagen-containing extracellular matrix, contractile fiber, I band, myofibril, and sarcomere were primarily enriched by ICM-specific genes. Analysis of molecular functions revealed the extracellular matrix structural constituent, glycosaminoglycan binding, heparin binding, integrin binding, and proteoglycan binding remarkedly related to ICM-specific genes (Figure 1E). KEGG analyses displayed that ICM-specific genes primarily participated in bladder cancer, cGMP-PKG signaling pathway, focal adhesion, HIF-1 signaling pathway, malaria, protein digestion and absorption, and proteoglycans in cancer (Figure 1F). Table 4 shows detailed information of the previous function enrichment results of ICM-specific genes.

**TABLE 2:** List of 62 upregulated ICM-specific genes.

Gene	logFC	AveExpr	t	FDR	Gene	logFC	AveExpr	t	FDR
ASPN	2.4402	8.3278	10.1871	7.41E-15	COL21A1	0.6819	7.5065	4.0706	0.00221
LUM	2.0637	8.1874	10.4394	2.50E-15	SYNPO2L	0.6757	8.2854	3.0047	0.03402
NPPA	1.7433	10.604	5.96104	3.86E-06	MFAP4	0.6752	7.4744	4.9059	0.00016
MXRA5	1.6391	7.8851	7.48832	6.20E-09	GJA1	0.6608	9.5343	4.4076	0.00082
HBB	1.484	10.454	5.2374	5.21E-05	HLTF	0.6579	7.1024	5.2503	5.07E-05
RPS4Y1	1.4123	9.1632	4.22763	0.001417	ZNF423	0.6536	5.8318	6.4674	5.32E-07
HBA1	1.334	10.048	4.86677	0.000187	SPARC	0.6531	9.3707	4.4771	0.00066
EIF1AY	1.2405	6.806	4.2239	0.001431	FHOD3	0.6487	9.4837	4.1558	0.00175
COL1A2	1.2123	8.9084	4.23877	0.001375	ITIH5	0.647	6.3416	6.6356	2.50E-07
COL3A1	1.1865	9.0459	4.11056	0.001997	APLNR	0.6439	7.9627	6.1372	2.00E-06
LOC100506558	1.1138	6.2107	7.72792	2.11E-09	HMGN2	0.6401	10.156	12.491	1.19E-19
OGN	1.045	5.0895	5.40275	3.00E-05	COL5A1	0.6334	7.021	3.0621	0.03001
PTN	1.0184	7.5082	8.3581	1.12E-10	C6	0.6315	8.9006	2.9926	0.03484
COL15A1	1.001	8.5994	5.94787	4.06E-06	CFH	0.629	7.1078	3.5601	0.00911
THBS4	0.949	9.5659	5.24705	5.10E-05	SERPINE2	0.6281	8.0896	2.821	0.04951
PPP1R3C	0.8637	10.307	5.28357	4.56E-05	PLXDC1	0.6279	6.1125	7.0112	4.62E-08
ISLR	0.8461	6.9838	7.69276	2.30E-09	KDM5D	0.6203	6.8871	3.8986	0.00361
LRRC17	0.8186	5.8017	5.0193	0.000112	PENK	0.6194	6.1351	4.051	0.00233
CXCL12	0.8003	8.5439	6.56103	3.55E-07	IDI2-AS1	0.6187	6.1857	4.02	0.00255
LTBP2	0.7885	8.9824	4.81306	0.000218	HLA-DPB1	0.6184	8.5418	4.1732	0.00166
IFI44L	0.788	6.0419	4.81949	0.000215	CTSK	0.6133	7.5298	6.1885	1.65E-06
KAL1	0.7838	6.5515	7.63272	3.02E-09	TPCN1	0.6125	8.4722	7.2974	1.34E-08
HTRA1	0.7743	8.8425	8.95584	5.05E-12	LHFP	0.6092	8.5637	6.1061	2.26E-06
ABCA8	0.7711	7.761	4.29693	0.001161	TRMT5	0.6075	9.3104	10.562	2.09E-15
ECM2	0.7657	6.9244	6.28461	1.15E-06	HSPA2	0.6026	7.5392	3.2734	0.01893
FRZB	0.7612	5.508	6.25454	1.24E-06	LRRC49	0.6002	8.2096	3.2838	0.01841
SLC27A6	0.7434	6.2606	3.77018	0.005242	EFEMP1	0.595	6.9869	2.8285	0.0488
SEPP1	0.7271	9.8408	4.44315	0.000729	CECR1	0.5931	8.5491	3.8985	0.00361
IFIT1	0.7123	6.7904	5.40383	3.00E-05	PDGFD	0.5872	7.5052	5.6166	1.37E-05
THBS2	0.6923	8.0236	3.54031	0.009599	PLA2G4C	0.5849	7.6363	4.757	0.00026
CRYM	0.6872	9.7867	3.88519	0.003742	FMOD	0.5847	7.7005	4.6802	0.00033

**TABLE 3:** List of 91 downregulated ICM-specific genes.

Gene	logFC	AveExpr	t	FDR	Gene	logFC	AveExpr	t	FDR
OGDH	−0.5843	9.1255	^−^3.4261	0.01275	ASNS	^−^0.7369	5.9882	^−^4.9005	0.00017
SRGN	^−^0.5856	8.1344	^−^2.8367	0.0482	CDK2AP2	^−^0.7454	7.112	^−^6.7748	1.36E-07
MAP2K1	^−^0.5867	8.0215	^−^6.7951	1.26E-07	RARRES1	^−^0.7475	5.094	^−^6.221	1.44E-06
ITGA5	^−^0.5903	8.1509	^−^5.3876	3.19E-05	ATP1A1	^−^0.7485	8.6287	^−^7.15	2.53E-08
ATP1B1	^−^0.5932	9.5308	^−^3.3459	0.01576	XIST	^−^0.7558	4.7123	^−^3.2627	0.01938
MIR3658	^−^0.595	6.2205	^−^4.0812	0.00216	KLHL41	^−^0.759	7.539	^−^3.6317	0.00759
PDLIM5	^−^0.5984	8.1782	^−^3.4965	0.01064	S100A9	^−^0.7608	7.4298	^−^2.9412	0.03887
PLEKHO1	^−^0.6079	6.5149	^−^5.6328	1.28E-05	TXNRD1	^−^0.7676	8.299	^−^6.6396	2.50E-07
DERL1	^−^0.623	5.7843	^−^7.2972	1.34E-08	CDC37L1	^−^0.7717	6.7248	^−^5.8272	6.09E-06
PNP	^−^0.6237	6.4093	^−^4.2904	0.00118	NPTX2	^−^0.7782	5.5116	^−^5.748	8.08E-06
NFIL3	^−^0.625	6.9906	^−^4.3035	0.00115	TGM2	^−^0.7893	7.2731	^−^5.3324	3.87E-05
TRAM1	^−^0.629	6.9059	^−^3.1049	0.02749	GPX3	^−^0.8047	10.754	^−^4.3172	0.0011
DNAJB1	^−^0.6346	7.4579	^−^5.0602	9.80E-05	NAMPT	^−^0.8144	7.7226	^−^5.2636	4.90E-05
FERMT2	^−^0.6383	6.5272	^−^4.7028	0.00032	FHL1	^−^0.819	10.222	^−^3.5997	0.00819
EDNRB	^−^0.6415	6.1138	^−^4.9645	0.00013	MAFF	^−^0.8217	5.3674	^−^4.7206	0.0003
POR	^−^0.6426	6.034	^−^6.1819	1.66E-06	PALLD	^−^0.8266	9.8876	^−^4.7074	0.00031
IRS2	^−^0.643	7.99	^−^5.7457	8.09E-06	IL1RL1	^−^0.8281	5.5744	^−^7.7171	2.12E-09
SLC38A1	^−^0.6435	8.7364	^−^3.2511	0.01991	DDX3X	^−^0.8301	6.6102	^−^5.4448	2.67E-05
THBS1	^−^0.6474	5.2093	^−^5.6927	1.02E-05	CLIC4	^−^0.8618	7.8744	^−^3.9074	0.00354
BCL6	^−^0.6479	6.8844	^−^7.1623	2.44E-08	HSP90AA1	^−^0.8736	10.134	^−^7.348	1.14E-08
ID1	^−^0.6589	8.4586	^−^3.0643	0.02991	LYVE1	^−^0.8896	6.6296	^−^4.79	0.00024
BAG3	^−^0.6607	9.4533	^−^5.7716	7.59E-06	TUBA3C	^−^0.8918	8.2595	^−^9.6639	1.09E-13
ATF3	^−^0.6638	6.7719	^−^3.2651	0.01931	SLCO4A1	^−^0.9151	6.898	^−^8.8407	8.68E-12
GNL3	^−^0.6657	5.6377	^−^4.4597	0.00069	MT1X	^−^0.9295	9.2248	^−^5.4333	2.79E-05
HSPD1	^−^0.666	8.7893	^−^5.8899	4.95E-06	SLC38A2	^−^0.9994	8.7183	^−^5.8034	6.67E-06
IFRD1	^−^0.6667	5.0369	^−^5.084	9.04E-05	FLNC	^−^1.038	10.538	^−^6.4569	5.50E-07
SLC22A5	^−^0.6687	6.4926	^−^6.2776	1.17E-06	MT1M	^−^1.0727	5.1601	^−^4.6912	0.00033
DDX39A	^−^0.6756	6.2528	^−^5.9856	3.52E-06	LMCD1	^−^1.0735	7.6006	^−^6.0458	2.91E-06
CSDC2	^−^0.6761	8.7018	^−^6.0214	3.13E-06	CCT2	^−^1.0842	7.1626	^−^7.75	1.98E-09
FADS3	^−^0.6835	7.3297	^−^7.1374	2.62E-08	SLC19A2	^−^1.0879	6.1056	^−^5.3269	3.94E-05
CD164	^−^0.6865	7.2865	^−^4.1015	0.00205	PDK4	^−^1.1839	7.7945	^−^2.9922	0.03484
MYH6	^−^0.6895	11.441	^−^4.3945	0.00086	PLA2G2A	^−^1.2347	8.069	^−^4.9951	0.00012
MYC	^−^0.6917	6.7113	^−^4.149	0.0018	CYP4B1	^−^1.279	5.8117	^−^5.5145	2.09E-05
ADH1B	^−^0.6984	5.523	^−^2.8413	0.0478	SERPINA3	^−^1.3068	7.2437	^−^9.9519	2.43E-14
TIMP4	^−^0.6997	6.7324	^−^5.374	3.35E-05	CNN1	^−^1.3164	8.2841	^−^7.7796	1.79E-09
AMD1	^−^0.6998	6.7461	^−^6.9097	7.36E-08	FCN3	^−^1.3399	6.9548	^−^10.709	1.36E-15
CDKN1A	^−^0.7026	8.6874	^−^6.2548	1.24E-06	PTX3	^−^1.3437	4.0272	^−^4.0754	0.00218
STAT3	^−^0.711	8.8764	^−^5.913	4.60E-06	ANKRD2	^−^1.3443	8.8635	^−^5.4933	2.19E-05
YBX3	^−^0.7166	9.9215	^−^4.854	0.00019	CD163	^−^1.3493	8.0004	^−^4.6114	0.00042
TIMP1	^−^0.7172	8.7828	^−^3.1852	0.02313	SERPINE1	^−^1.3766	6.856	^−^4.6951	0.00032
MTHFD2	^−^0.7185	6.5773	^−^4.0816	0.00216	GLUL	^−^1.4711	7.5375	^−^7.2156	1.91E-08
PCNT	^−^0.7189	7.8582	^−^6.6408	2.50E-07	NRAP	^−^1.488	8.459	^−^3.7919	0.00489
GLRX2	^−^0.7243	7.016	^−^8.2261	2.12E-10	FKBP5	^−^1.4929	5.9928	^−^7.3334	1.19E-08
TNFRSF11B	^−^0.7285	4.5461	^−^4.1472	0.00181	HOPX	^−^1.498	6.6634	^−^8.1784	2.55E-10
FOSL1	^−^0.7298	5.7306	^−^5.2025	5.94E-05	MYOT	^−^2.2659	6.2321	^−^10.289	4.88E-15
EIF1AX	^−^0.7367	6.5696	^−^5.9261	4.40E-06					

**TABLE 4:** Detailed information of function enrichment results of ICM-specific genes.

Description	GeneRatio	BgRatio	*p*-value	Adjusted p	q-value	Count
Extracellular matrix organization	17/146	368/18670	4.66E-09	7.10E-06	5.88E-06	17
Extracellular structure organization	17/146	369/18670	4.85E-09	7.10E-06	5.88E-06	17
Cellular response to external stimulus	15/146	339/18670	6.99E-08	6.82E-05	5.65E-05	15
Response to mechanical stimulus	12/146	210/18670	9.84E-08	7.20E-05	5.96E-05	12
Response to transforming growth factor beta	12/146	255/18670	7.94E-07	0.000464	0.000385	12
Collagen-containing extracellular matrix	29/144	406/19717	1.14E-20	2.95E-18	2.48E-18	29
Contractile fiber	14/144	234/19717	1.87E-09	2.42E-07	2.03E-07	14
Sarcomere	13/144	204/19717	3.41E-09	2.93E-07	2.46E-07	13
I band	11/144	143/19717	8.13E-09	5.25E-07	4.41E-07	11
Myofibril	13/144	224/19717	1.05E-08	5.42E-07	4.56E-07	13
Extracellular matrix structural constituent	16/141	163/17696	2.28E-13	9.38E-11	7.79E-11	16
Heparin binding	9/141	169/17696	8.50E-06	0.001047	0.00087	9
Proteoglycan binding	5/141	36/17696	9.24E-06	0.001047	0.00087	5
Integrin binding	8/141	132/17696	1.07E-05	0.001047	0.00087	8
Glycosaminoglycan binding	10/141	229/17696	1.52E-05	0.001047	0.00087	10
Protein digestion and absorption	8/82	103/8105	8.48E-06	0.001917	0.001616	8
Malaria	5/82	50/8105	0.000139	0.015705	0.01324	5
Proteoglycans in cancer	9/82	205/8105	0.000211	0.015869	0.013378	9
Bladder cancer	4/82	41/8105	0.000741	0.034691	0.029246	4
HIF-1 signaling pathway	6/82	109/8105	0.000788	0.034691	0.029246	6
Focal adhesion	8/82	201/8105	0.000921	0.034691	0.029246	8
cGMP–PKG signaling pathway	7/82	167/8105	0.001444	0.046626	0.039308	7

### Analysis of Interactions Between ICM-Specific Genes

**FIGURE 2:**
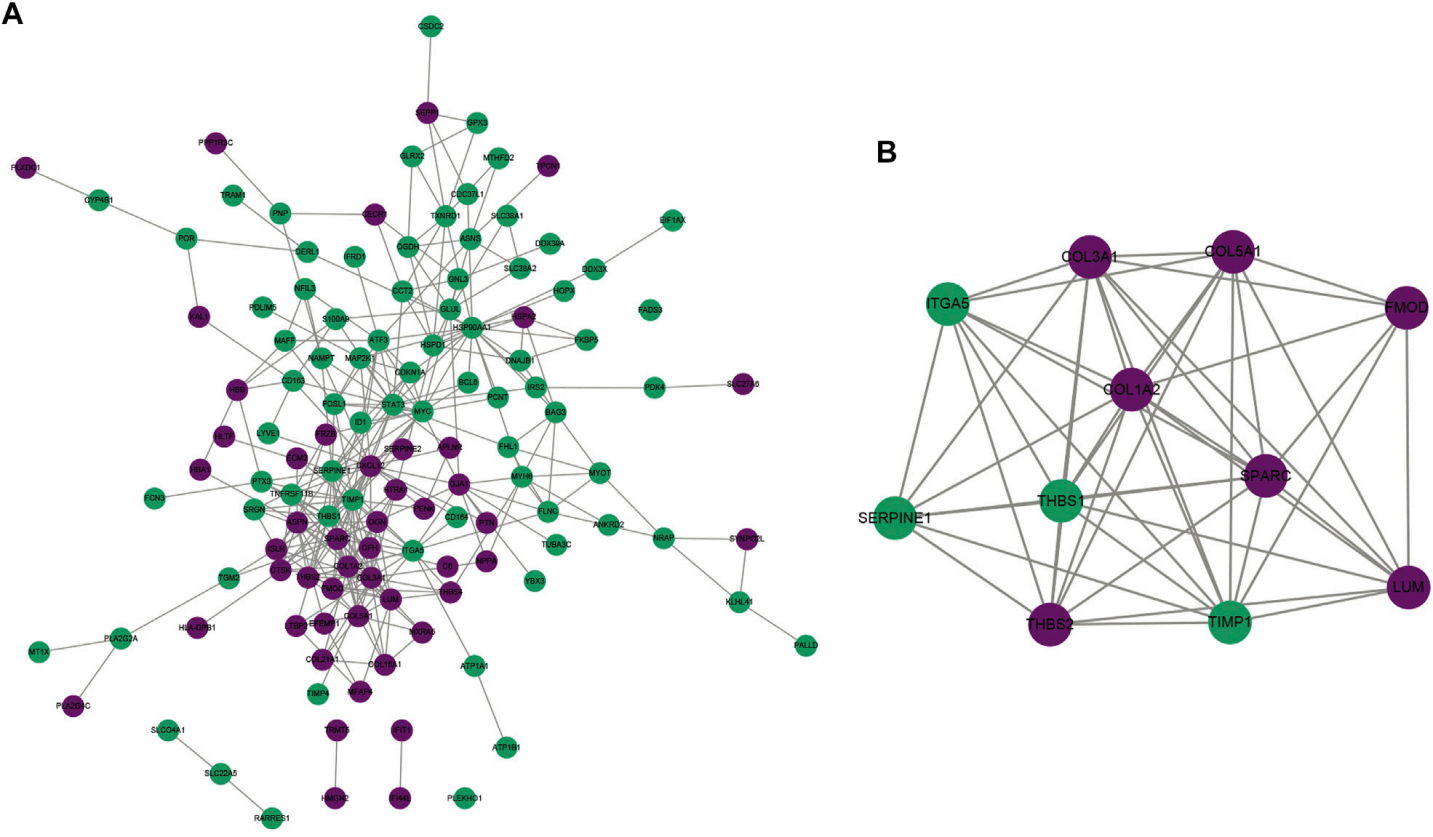
Analysis of interactions between ICM-specific genes. **(A)** The PPI network was conducted for uncovering the interactions between ICM-specific genes. **(B**) The most prominent module was determined *via* MCODE plug-in. Upregulated ICM-specific genes are marked purple, whereas downregulated ICM-specific genes are marked green.

Through the STRING project, we conducted the PPI network of ICM-specific genes. ICM-specific genes with high expression were marked with purple color, while ICM-specific genes with low expression were marked with blue color. There were 322 interaction pairs in the PPI network, uncovering the close interactions between ICM-specific genes (Figure 2A). For figuring out the most prominent module of this complex network, this study presented the MCODE plug-in analyses. This module contained seven upregulated ICM-specific genes (COL3A1, COL1A2, COL5A1, SPARC, FMOD, LUM, and THBS2) and four downregulated genes (ITGA5, THBS1, SERPINE1, and TIMP1), as depicted in Figure 2B.

### Selection of Candidate ICM-Specific Genes Through Integration of Three Algorithms: LASSO, SVM, and Random Forest

**FIGURE 3:**
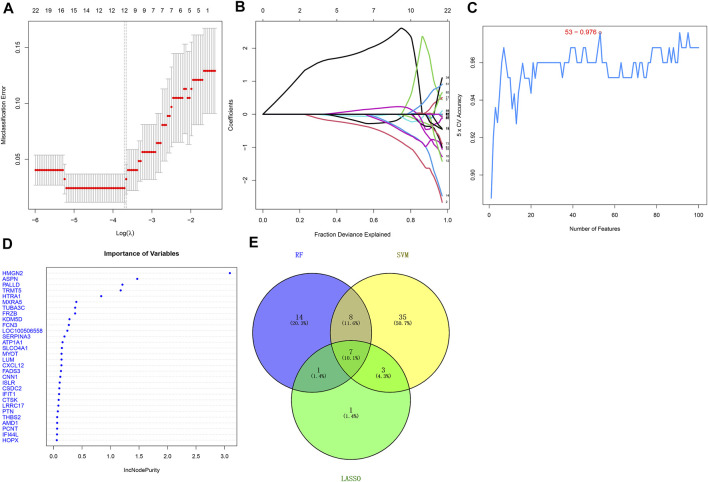
Selection of candidate ICM-specific genes through integration of three algorithms: LASSO, SVM, and random forest. **(A)** LASSO coefficient spectrum of ICM-specific genes across ICM samples. Coefficient distribution diagram was generated regarding logarithmic (λ) sequences. **(B)** Selection of the optimal variables for ICM within the LASSO model (λ). **(C)** Screening characteristic genes using the SVM method. **(D)** Random forest was adopted for selecting important variables. **(E)** Venn diagram shows candidate ICM-specific genes through integration of results derived from three algorithms.

This study adopted three feature selection algorithms including LASSO, SVM, and random forest for identifying candidate ICM-specific genes. By using the LASSO method, we identified 12 candidate variables containing HMGN2, FCN3, TRMT5, LUM, ASPN, HOPX, CNN1, GLUL, PCNT, NPPA, THBS1, and GPX3 (Figures 3A,B). The SVM method uncovered 53 feature genes (Figure 3C). Meanwhile, 23 important variables were selected using the random forest method (Figure 3D). Following the integration of results derived from three algorithms, seven candidate ICM-specific genes were finally identified, including ASPN, TRMT5, LUM, FCN3, CNN1, PCNT, and HOPX (Figure 3E).

### Combination of Candidate ICM-Specific Genes as a Reliable Diagnostic Model of ICM

**FIGURE 4:**
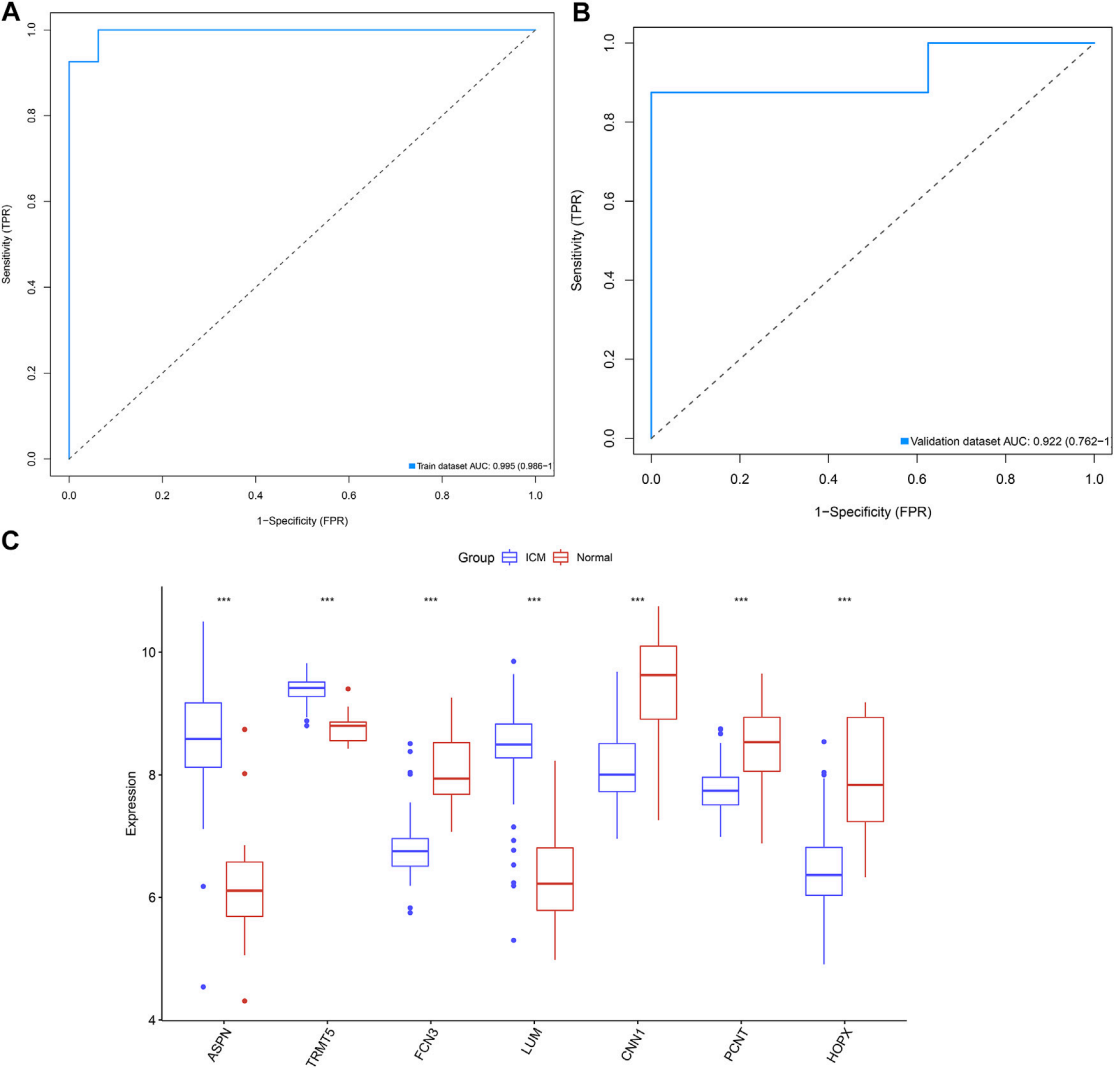
Combination of candidate ICM-specific genes as a reliable diagnostic model of ICM. **(A)** ROC curves were conducted for evaluations of the diagnostic potential of this combination of candidate ICM-specific genes in the GSE5406 dataset. **(B)** Verification of the diagnostic value of this combination of candidate ICM-specific genes in the GSE46224 dataset. **(C)** Box plots shows the deregulated expressions of these candidate ICM-specific genes in ICM and controls. ***p < 0.001.

ROC curves were conducted for investigations of the diagnostic efficacy of this combination of candidate ICM-specific genes in ICM. As depicted in Figure 4A, the AUC of this combined gene model was 0.995 in the GSE5406 dataset, confirming the excellent diagnostic efficacy in ICM. Additionally, the GSE46224 dataset was adopted for verification of this model. As a result, AUC was 0.922, indicative of its favorable predictive potential (Figure 4B). The expressions of candidate ICM-specific genes were investigated in ICM and control myocardial tissues in the GSE5406 dataset. As a result, ASPN, TRMT5, and LUM expressions were upregulated, while FCN3, CNN1, PCNT, and HOPX expressions were declined in ICM compared with controls (Figure 4C).

### Characterization of Immune Cell Infiltrates in ICM and Control Myocardial Tissues

**FIGURE 5:**
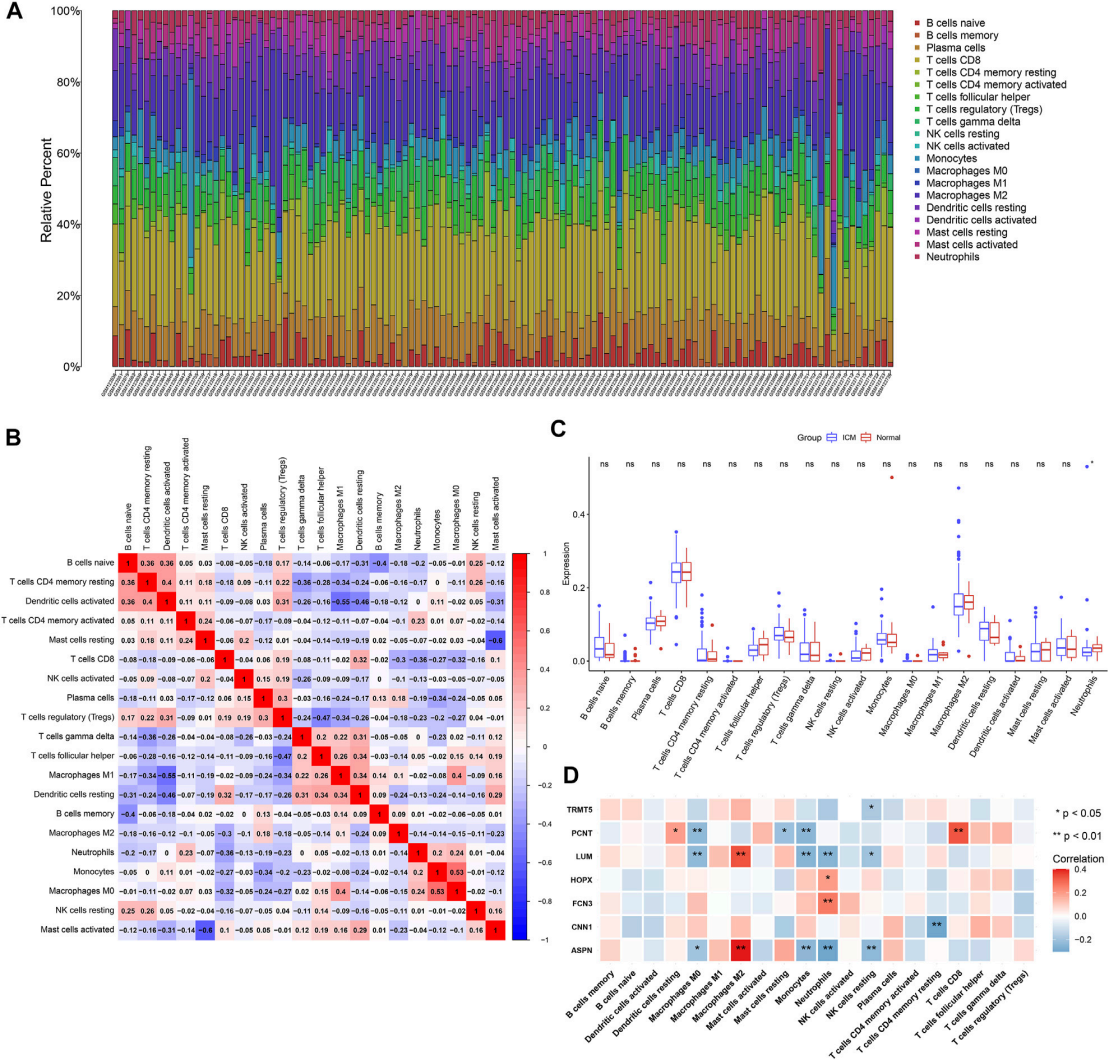
Landscape of immune cell infiltrates in ICM and interactions of candidate ICM-specific genes with immune cell infiltrates. **(A)** Stacked graphs depict the percentages of 22 immune cell subpopulations in ICM myocardial tissues. **(B)** Heatmap shows the interactions of diverse immune cell subpopulations in ICM myocardial tissues. The deeper the red, the stronger the positive correlation. The darker the blue, the stronger the negative correlation. **(C)** Box plots depict the differences in immune cell infiltrates in ICM and normal myocardial tissues. **(D)** Heatmap visualizes the interactions of candidate ICM-specific genes with immune cell infiltrates in ICM. *p < 0.05; **p < 0.01; ns: not significant.

Using the CIBERSORT algorithm, we estimated the infiltrations of immune cell subpopulations in ICM myocardial tissues. Figure 5A depicts the landscape of 22 immune cell subpopulations across ICM. We also presented investigations about the interactions between immune cell subpopulations. In Figure 5B, there were close interplays between diverse immune cell subpopulations across ICM. In particular, compared with controls, neutrophil presented declined infiltrations in ICM (Figure 5C).

### Interactions of Candidate ICM-Specific Genes With Immune Cell Infiltrates

This study analyzed the interactions of candidate ICM-specific genes with immune cell infiltrates across ICM myocardial tissues. Our data suggested that ASPN and LUM expressions were positively associated with M2 macrophage while negatively correlated to M0 macrophages, monocytes, neutrophils, and resting NK cells (Figure 5D). CNN1 expressions displayed negative correlations to CD4 memory resting T cells as well as FCN3 and HOPX3 expressions exhibited positive associations with neutrophils. PCNT expressions were positively associated with the resting dendritic cells and CD8 T cells while negatively correlated to M0 macrophages, resting mast cells, and monocytes. Additionally, TRMT5 expressions presented negative interactions with resting NK cells.

### Verification of Expressions of Candidate ICM-Specific Genes

**FIGURE 6:**
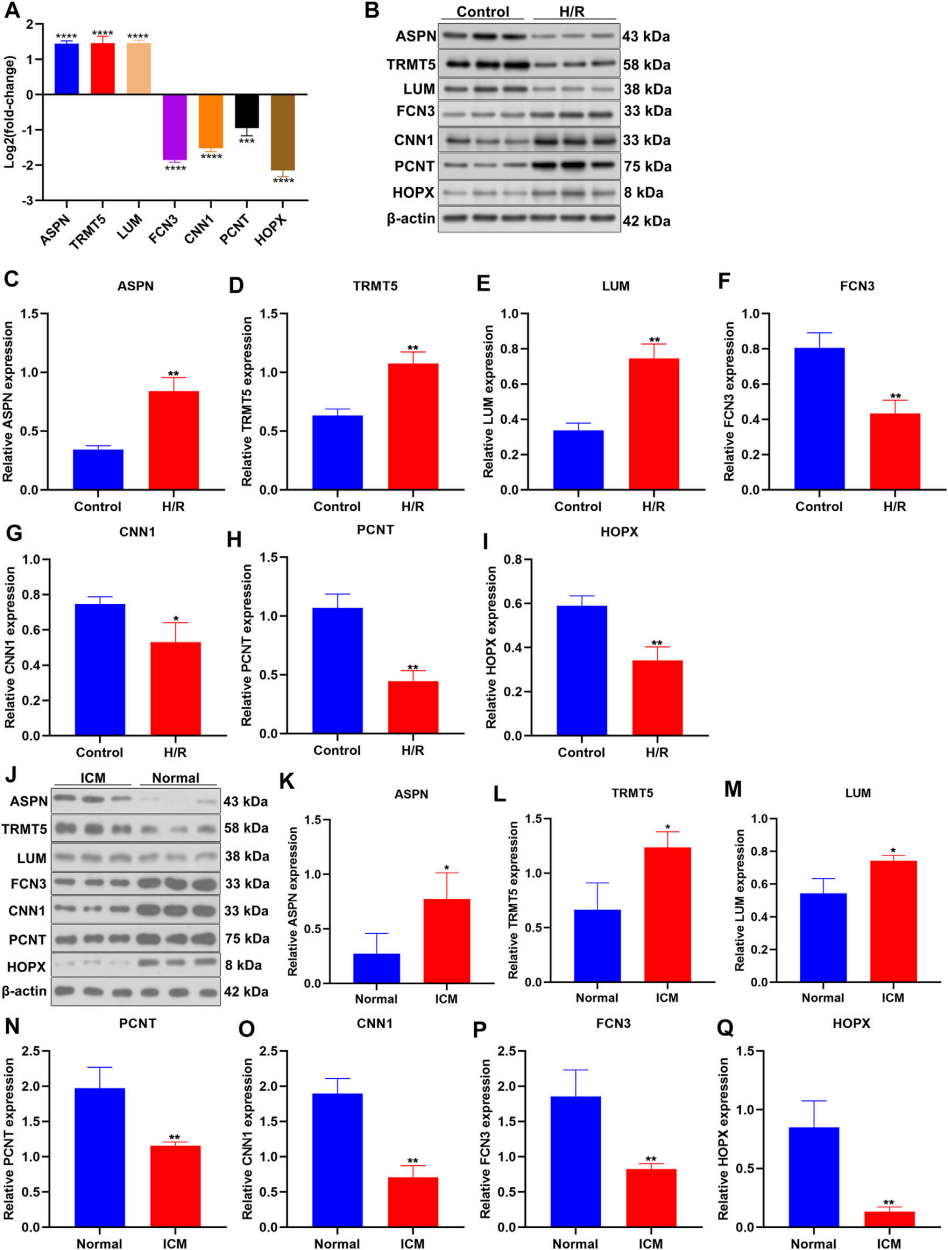
Verification of expressions of candidate ICM-specific genes. **(A)** The mRNA expressions of candidate ICM-specific genes including ASPN, TRMT5, LUM, FCN3, CNN1, PCNT, and HOPX were investigated in H/R-stimulated H9C2 cells and control cells with RT-qPCR. **(B–I)** Deregulated expressions of ASPN, TRMT5, LUM, FCN3, CNN1, PCNT, and HOPX were examined in H/R-stimulated H9C2 cells and control cells by Western blotting. **(J–Q)** Abnormal expressions of ASPN, TRMT5, LUM, FCN3, CNN1, PCNT, and HOPX were examined in three human ICM and control left ventricular myocardial tissues by Western blotting. *n* = 3. *p*-values were estimated using Student’s test. *p < 0.05; **p < 0.01; ***p < 0.001; ****p < 0.0001.

Herein, we established H/R-stimulated ICM cellular models. By RT-qPCR, we conducted investigations of the mRNA expressions of candidate ICM-specific genes including ASPN, TRMT5, LUM, FCN3, CNN1, PCNT, and HOPX. Figure 6A demonstrates that ASPN, TRMT5, and LUM mRNAs presented the upregulations in H/R-stimulated H9C2 cells compared to controls. In contrast, FCN3, CNN1, PCNT, and HOPX expressions were declined in H/R-stimulated H9C2 cells than controls. Additionally, Western blotting uncovered the elevated expressions of ASPN, TRMT5, and LUM in H/R-stimulated H9C2 cells compared with control cells (Figures 6B–E). Meanwhile, lower FCN3, CNN1, PCNT, and HOPX expressions were observed in H/R-stimulated H9C2 cells compared with controls (Figures 6F–I). We collected three ICM and three healthy left ventricular myocardial tissues. Western blotting confirmed the upregulations of ASPN, TRMT5, and LUM as well as the downregulations of FCN3, CNN1, PCNT, and HOPX in ICM compared with healthy left ventricular myocardial tissues (Figures 6J–Q). These data confirmed the deregulated expressions of candidate ICM-specific genes in ICM.

### TRMT5 Deficiency Enhances Mitochondrial Membrane Potential of H/R-Stimulated H9C2 Cells

**FIGURE 7:**
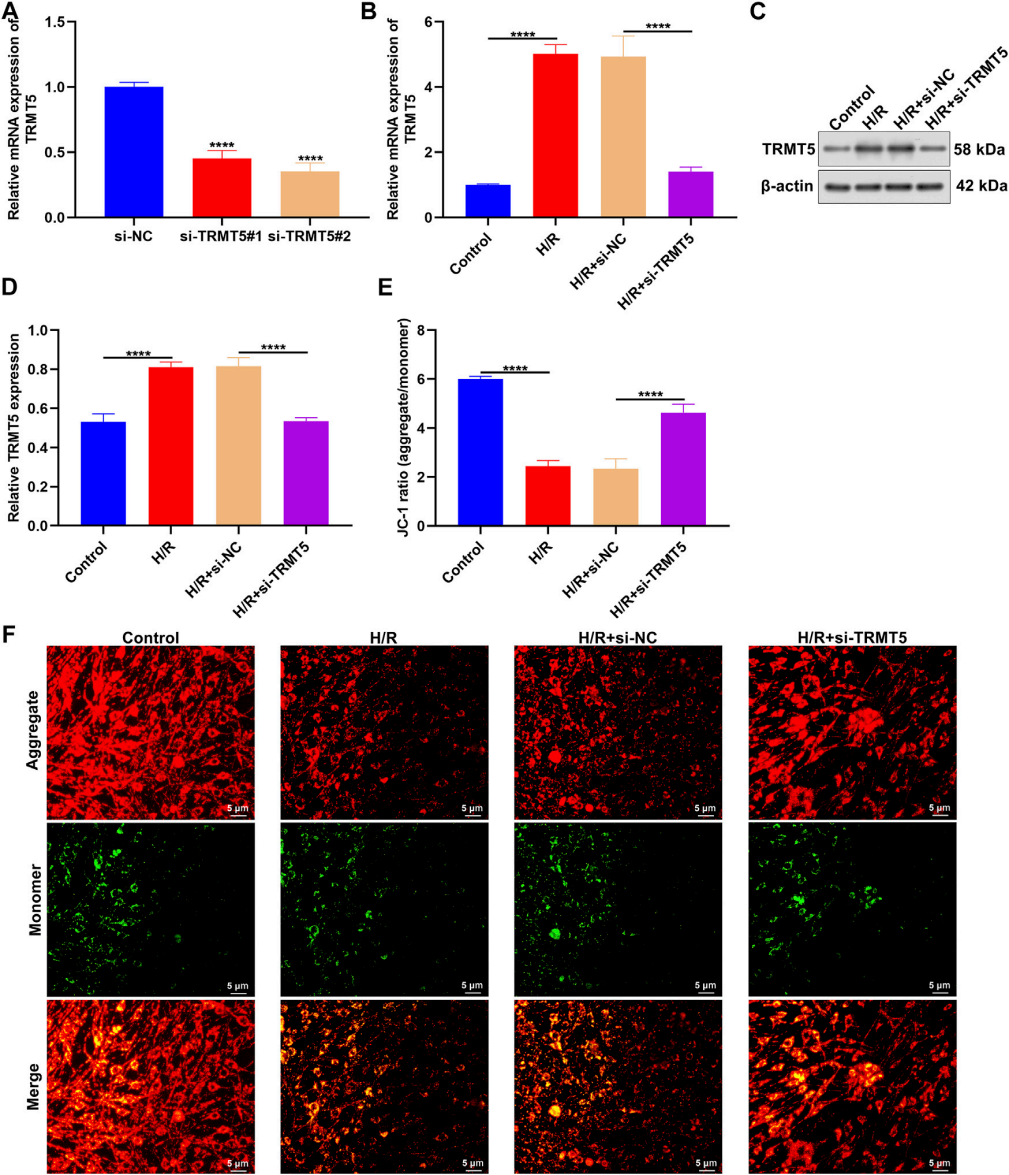
TRMT5 deficiency enhances mitochondrial membrane potential of H/R-stimulated H9C2 cells. **(A)** The mRNA expressions of TRMT5 were examined in H9C2 cells with specific siRNAs against TRMT5 transfections. **(B–D)** RT-qPCR and Western blotting was presented for quantification of TRMT5 expressions in H9C2 cells with H/R stimulation and si-TRMT5 transfections. **(E,F)** JC-1 staining was adopted for the determination of mitochondrial membrane potential of H9C2 cells with H/R stimulation and si-TRMT5 transfections. The ratio of red (aggregate) to green (monomer) fluorescence represented mitochondrial membrane potential. Magnification, ×200; scale bar, 5 μm. *n* = 3. *p*-values were estimated using ANOVA with Tukey’s post hoc test. ****p < 0.0001.

Among candidate ICM-specific genes, the role of TRMT5 in ICM has not yet been reported. Therefore, we presented an in-depth investigation about the implications of TRMT5 in ICM progression. TRMT5 expressions were first deficient in H9C2 cells through transfections of si-TRMT5. In Figure 7A, TRMT5 deficiency triggered by si-TRMT5 was confirmed in H9C2 cells. Intriguingly, TRMT5 expressions presented upregulation in H/R-stimulated H9C2 cells compared to controls (Figures 7B–D). Nevertheless, si-TRMT5 remarkedly reduced the expressions of TRMT5 stimulated by H/R. JC-1 staining and was employed for the evaluation of mitochondrial membrane potential. In Figures 7E,F, the reduced ratios of red/green fluorescence were noticed in H/R-exposed H9C2 cells than controls, which indicated that the mitochondrial membrane potential of H9C2 cells was decreased following H/R stimulation. Additionally, the ratios of red/green fluorescence were elevated in H/R-exposed H9C2 cells with si-TRMT5 transfections in comparison to H/R-exposed H9C2 cells. This indicated that TRMT5 deficiency enhanced the mitochondrial membrane potential of H/R-stimulated H9C2 cells.

### TRMT5 Deficiency Alleviates H/R-Triggered H9C2 Cell Apoptosis

**FIGURE 8:**
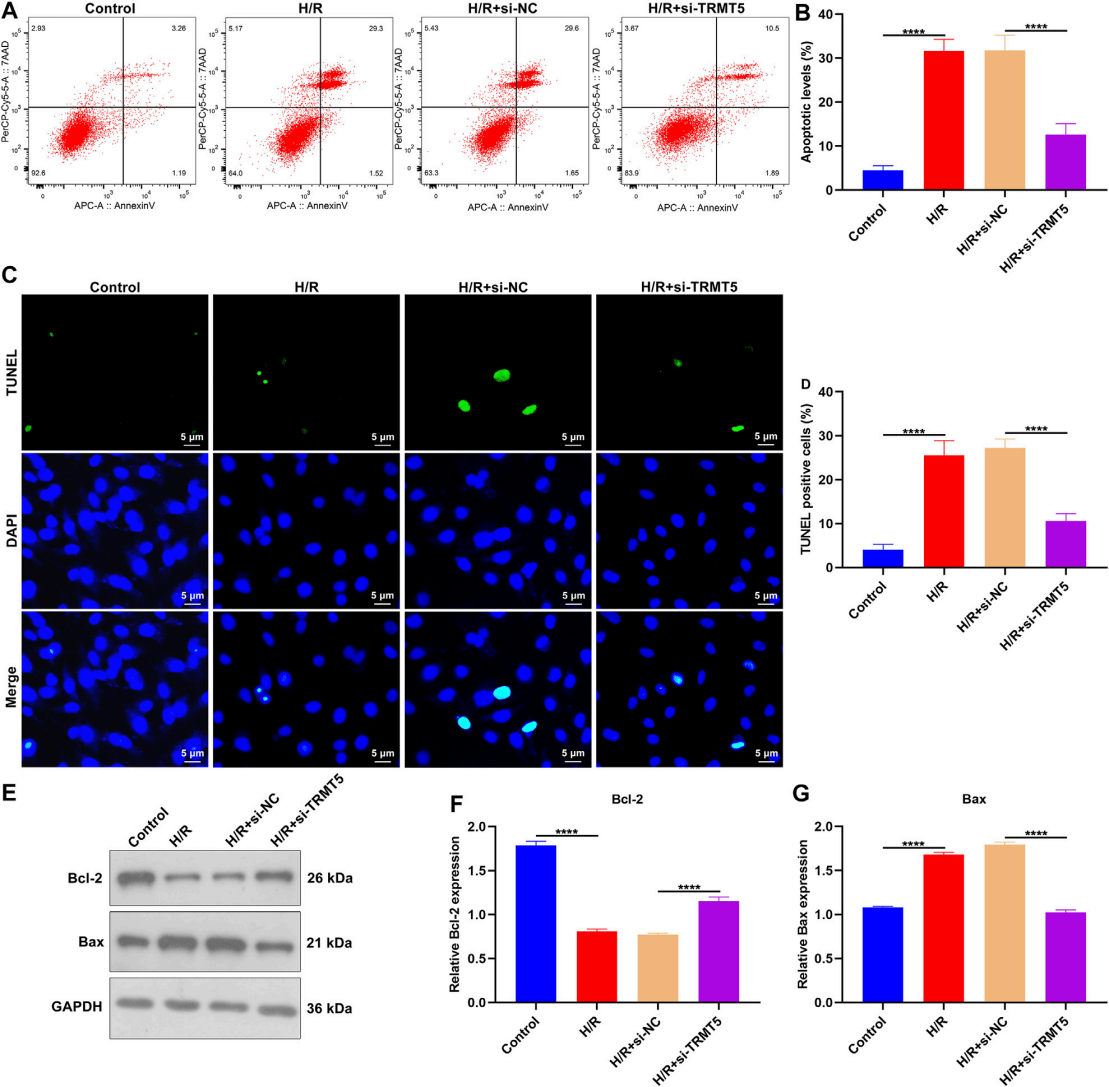
TRMT5 deficiency alleviates H/R-triggered H9C2 cell apoptosis. **(A,B)** Apoptotic H9C2 cells were estimated by flow cytometric analyses following H/R exposure and TRMT5 deficiency. **(C,D)** Apoptosis was tested utilizing TUNEL staining in H9C2 cells with H/R exposure and TRMT5 deficiency. Magnification, ×200; scale bar, 5 μm. **(E–G)** Western blotting was used for detecting the expression of Bcl-2 and Bax in H9C2 cells with H/R exposure as well as TRMT5 deficiency. N = 3. *p*-values were estimated using ANOVA with Tukey’s post hoc test. ****p < 0.0001.

Flow cytometric analyses showed the increased apoptotic levels of H/R-stimulated H9C2 cells than controls (Figures 8A,B). Nevertheless, the apoptosis of H9C2 cells was remarkedly declined following TRMT5 deficiency. Additionally, we presented TUNEL assay for investigations of cardiomyocyte apoptosis. More TUNEL-positive cells were detected in H/R-exposed H9C2 cells (Figures 8C,D). But TRMT5 deficiency reduced the ratios of TUNEL-positive H/R-exposed H9C2 cells. We also noted that the Bcl-2 expression was downregulated, while Bax expression was upregulated in H/R-exposed H9C2 cells (Figures 8E–G). However, TRMT5 blockage significantly reversed the expression of Bcl-2 and Bax in H/R-exposed H9C2 cells. Hence, TRMT5 deficiency alleviated H/R-triggered H9C2 cell apoptosis.

## Discussion

Deregulated expression of genes may trigger ICM progression and dismal clinical prognosis. This study detected 153 ICM-specific genes; among them, 62 presented upregulation, while 91 presented downregulation in ICM myocardium tissues. In particular, these genes participated in ICM-relevant processes and signals like extracellular matrix and structure organization and HIF-1 signaling pathway. The data highlighted the underlying implications of ICM-specific genes during ICM progression.

Through combination strategies of LASSO, SVM, and random forest algorithms, we eventually identified seven reliable candidate ICM-specific genes (ASPN, TRMT5, LUM, FCN3, CNN1, PCNT, and HOPX). ROC curves confirmed that the combination of previous seven candidate ICM-specific genes acted as an accurate diagnostic tool for ICM patients. ASPN, TRMT5, and LUM expressions presented the upregulations in ICM than controls. In contrast, FCN3, CNN1, PCNT, and HOPX expressions were declined in ICM than controls. Previously, ASPN protein displayed increased expressions in ICM left ventricular tissues that are highly correlated to left ventricular ejection fraction (Zhang et al., 2021). Moreover, ASPN expressions are upregulated in cardiac remodeling mouse models (Wang et al., 2019). The upregulations are proposed in the myocardium of diabetic mice and in glycated low-density lipoprotein-triggered cardiomyocytes (Li et al., 2019). Moreover, ASPN triggers the apoptosis of H9C2 cardiomyocytes. The downregulation of circulating FCN3 levels is correlated to advanced heart failure and outcomes (Prohászka et al., 2013). CNN1 suppresses dilated cardiomyopathy progression in mice *via* εPKC signaling (Lu et al., 2014). Moreover, it modulates TNF-α-triggered apoptosis of vascular endothelial cells (Zhang et al., 2016). Single-cell sequence analyses of cardiac differentiation from human pluripotent stem cells uncover HOPX-mediated maturation of cardiomyocytes (Friedman et al., 2018). The previous evidence suggested the functions of these candidate ICM-specific genes in ICM progression.

ICM acts as a state of chronic immune system activation (Sweet et al., 2018; Bansal et al., 2019; Chelko et al., 2019). Here, we quantified immune cell infiltrates across ICM myocardial tissues using the CIBERSORT method. ICM myocardial tissues had declined infiltrations of neutrophils than controls, indicating that deregulated neutrophils might participate in ICM pathogenesis. Innate immune response and leukocyte recruitment modulate inflammation and healing, playing a critical function in acute myocardial infarction (Eghbalzadeh et al., 2019). Neutrophils participate in the healing following myocardial infarction by polarizing macrophages into a repair phenotype (Horckmans et al., 2017). Deregulated expressions of ASPN, TRMT5, LUM, FCN3, CNN1, PCNT, and HOPX were in relation to diverse immune cell subpopulations across ICM myocardial tissues. This indicated that the previous candidate ICM-specific genes might modulate inflammatory response during ICM progression. In particular, we paid close attention to the implications of TRMT5 in ICM. Experimental evidence showed that H/R stimulation triggered the upregulation of TRMT5 in H9C2 cells (Powell et al., 2015). TRMT5 deficiency enhanced mitochondrial membrane potential and reduced apoptosis in H/R-exposed H9C2 cells. Thus, TRMT5 possessed the potential as a therapeutic target against ICM. Nevertheless, this study presented several disadvantages. The diagnostic potential of this combination of candidate ICM-specific genes will be verified in larger ICM cohorts. Additionally, biological functions and mechanisms involving these ICM-specific genes will be investigated through experiments. Conclusion

This study proposed candidate ICM-specific genes through combination strategies of LASSO, SVM, and random forest algorithms. Our in-depth analyses uncovered that ASPN, TRMT5, LUM, FCN3, CNN1, PCNT, and HOPX might be exploited as reliable diagnostic signatures of ICM. Hence, our findings offered an underlying basis for understanding the etiology and mechanisms of ICM as well as therapeutic targets for clinic therapy.

## Data Availability

The original contributions presented in the study are included in the article/supplementary material; further inquiries can be directed to the corresponding authors.

## Abbreviations

ICM, ischemic cardiomyopathy; GEO, gene expression omnibus; limma, linear models for microarray data; FC, fold change; GO, gene ontology; KEGG, kyoto encyclopedia of genes and genomes; PPI, protein–protein interaction; STRING, search tool for the retrieval of interacting genes/proteins; MCODE, molecular complex detection; LASSO, least absolute shrinkage and selection operator; SVM, support vector machine; ROC, receiver operating characteristic; AUC, area under the curve; CIBERSORT, cell type identification by estimating relative subsets of rna transcripts; RT-qPCR, real-time quantitative polymerase chain reaction; h/r, hypoxia/reoxygenation; TUNEL, terminal deoxynucleotidyl transferase dUTP nick-end labeling.
